# *In situ* synchrotron study of electromigration induced grain rotations in Sn solder joints

**DOI:** 10.1038/srep24418

**Published:** 2016-04-18

**Authors:** Hao Shen, Wenxin Zhu, Yao Li, Nobumichi Tamura, Kai Chen

**Affiliations:** 1 Center for Advancing Materials Performance from the Nanoscale (CAMP-Nano), State Key Laboratory for Mechanical Behavior of Materials, Xi’an Jiaotong University, Xi’an, Shaanxi 710049 China; 2 Advanced Light Source, Lawrence Berkeley National Laboratory, Berkeley, California 94720, USA

## Abstract

Here we report an *in situ* study of the early stage of microstructure evolution induced by electromigration in a Pb-free β-Sn based solder joint by synchrotron polychromatic X-ray microdiffraction. With this technique, crystal orientation evolution is monitored at intragranular levels with high spatial and angular resolution. During the entire experiment, no crystal growth is detected, and rigid grain rotation is observed only in the two grains within the current crowding region, where high density and divergence of electric current occur. Theoretical calculation indicates that the trend of electrical resistance drop still holds under the present conditions in the grain with high electrical resistivity, while the other grain with low resistivity reorients to align its *a*-axis more parallel with the ones of its neighboring grains. A detailed study of dislocation densities and subgrain boundaries suggests that grain rotation in β-Sn, unlike grain rotation in high melting temperature metals which undergo displacive deformation, is accomplished via diffusional process mainly, due to the high homologous temperature.

The deleterious electromigration (EM) phenomenon becomes more severe with the current trend of miniaturization of electronic devices, for both interconnect lines and solder joints^1^,^2^. Tremendous efforts have been made to study not only the formation of voids at the cathode end and extrusions at the anode end, but also the microstructure evolution of the materials induced by the high electric current density at even earlier stage, well before the failure of the electronic devices. Grain rotation has been reported in all the metals used in interconnect components, including Al^3^,^4^,^5^, Cu^6^,^7^, and β-Sn^8^,^9^. In face-centered cubic (FCC) metals Al and Cu, rotation of several degrees is realized via the generation of geometrically necessary dislocations (GNDs) and geometrically necessary boundaries (GNBs) under the stress gradient created by EM. β-Sn has a body-centered tetragonal (BCT) crystal structure and its electrical conductivity^10^ and self-diffusivity^11^ are anisotropic with greater values along *a*- and *b*-axes than along *c*-axis. In β-Sn strip lines, grain rotation of tens of degrees is observed accompanied with grain growth in a mechanism to realign the high electrical conductivity crystal direction with the electric current flow. The resulting large resistance decrease can cause electric current fluctuations and impact the reliability of the Sn components. However, it is not yet clear if similar phenomena also take place in Pb-free solder joints, which are mainly composed of β-Sn. On one hand, because the electric current direction and density in a solder joint are more inhomogeneous than in strips due to the complicated line-to-bump configuration in flip chips, current crowding effect has to be taken into account^12^,^13^ and therefore the stress state in the current crowding region is expected to be more inhomogeneous. On the other hand, hillock growth induced by EM has been observed under scanning electron microscope (SEM)^14^, and elastic compressive transient stress has been detected near the anode current crowding region and quantitatively measured^15^, so it is of interest to understand how the materials undergo plastic deformation when the stress reaches yield values. If grain growth observed in the Sn strips is attributed to the thermomechanical deformation induced by EM, it is interesting to ask what triggers the growth at the initial stage.

Here we study the early stage of the evolution of crystal orientation in β-Sn grains induced by high density of electric current in a Pb-free solder joint using synchrotron polychromatic X-ray microdiffraction (μXRD). Under the experimental conditions applied in this study, grain growth is not observed, and rotation of approximately half a degree is detected in the grains within the current crowding region only. The investigation of the evolution of electrical resistivity leads us to attribute the driving force of grain rotation to the minimization of electrical resistance. The study of the evolution of Laue diffraction peak shape shows no generation of GNDs and GNBs, indicating a rigid rotation mechanism in stark contrast to the dislocation slip mediated rotation in FCC Al and Cu metals and grain growth mediated rotation in Sn strip lines. The grain rotation is achieved via diffusional deformation induced by the unbalanced atomic diffusion in the current crowding region.

## Results

**Figure 1:**
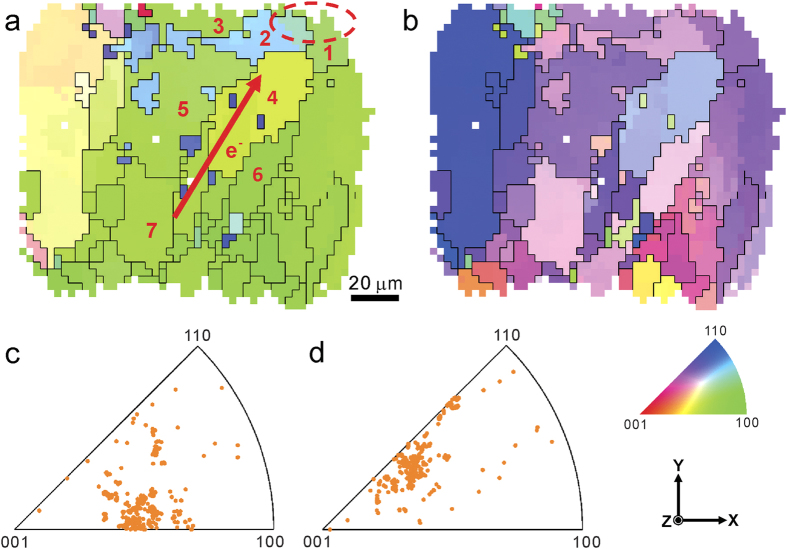
Crystal orientation distribution in the solder joint before the EM test obtained from μXRD. (**a**,**b**) The orientation maps and their inverse pole figures of the in-plane **X**- and **Y**-directions, respectively. Electric current flow direction as well as the current crowding region are marked, and 7 grains are numbered for detailed study.

The cross-section of a Pb-free Sn-based solder joint was raster scanned with μXRD before and during the EM test, and 11 scans were made in total within a 43 h period. More experimental details are given in the Methods section. To express the crystal orientation, a Cartesian coordinate system **O-XYZ** was established, with its **X-** and **Y-**axes parallel with the horizontal and vertical scanning directions, respectively, and its **Z-**axis perpendicular to the sample surface (shown in Fig. 1). Two dimensional orientation maps were generated by indexing all the Laue diffraction patterns. Fig. 1a,b show the crystal orientation distributions of the cross-section of the Sn solder joint along the **X**- and **Y**-scanning directions, respectively, before the EM test. Black curves in Fig. 1a,b display the grain boundaries, which are defined as disorientation angles greater than 5° between two adjacent pixels, and herein 34 grains are counted in the maps^16^. Most of the grains, especially in the middle and right half of the solder joint, show green and purple color in Fig. 1a,b, respectively, indicating preferential crystal orientation. This preferential texture is also evident from the inverse pole figures, shown in Fig. 1c,d.

In the EM experiment, the electric current flows from the bottom left to the top right of the solder joint as indicated by the red arrow, and the upper-right corner (red ellipse in Fig. 1a) is the current crowding region, where the current density is about one order of magnitude higher than other regions and where high current density gradient exists^12^. Seven grains in the electron wind force impacted region are selected and numbered for detailed study and their microstructures are tracked throughout the 11 scans. The angle between the *c*-axis of the selected grains and the electric current direction is 45° or higher (listed in Table 1), which suggests that in this specimen, the self-diffusion of Sn plays a more important role than the fast diffusion of Cu in Sn^17^.

**Figure 2:**
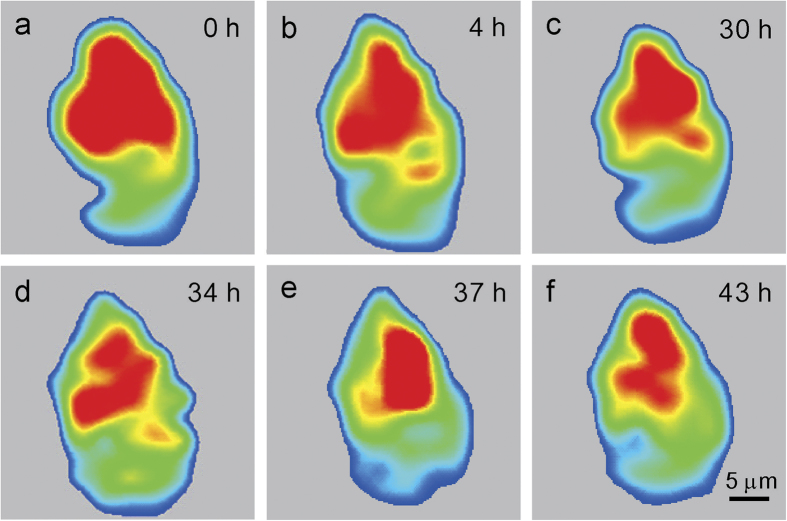
Shape mapping of Grain 1. By plotting the spatial distribution of (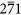) Laue peak intensity from each μXRD scan, the morphology and size of Grain 1 are mapped as a function of EM time. By this approach, three positions close to the center of the grain are pinpointed in each scan to investigate the orientation evolution.

The orientation maps obtained from each scan are carefully compared to monitor the orientation, shape, and size evolution of the selected grains. The shape evolution of the grain labelled “Grain 1” is plotted via intensity maps. Such intensity maps are obtained by first indexing all the Laue patterns from this crystal grain, and then tracking the intensity of a specific diffraction peak. It is noted that this peak should show up at the same position on all the Laue patterns in this grain. Here we choose the (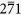) peak. The integrated intensity of peak from all patterns is recorded, and a 2D contour map is plotted with the color coding intensity values (Fig. 2). It can be seen that the morphology and size of Grain 1 remain unchanged under the high density electric current stresses within the spatial resolution (3 μm, determined by the scanning step size) of this study. The same methodology is applied to all the other grains. No monotonic change of grain size is observed, and the size fluctuation of all the tracked 7 grains is less than 2 pixels, caused by sample drift and temperature fluctuation during the measurement, suggesting that no obvious grain growth is triggered by the electric current. The color of all the 7 grains in the orientation maps remains unchanged through the 11 scans, indicating no dramatic rotation of tens of degrees as what was reported in the Sn strips^8^ or appearance of cyclic twinning^17^.

**Figure 3:**
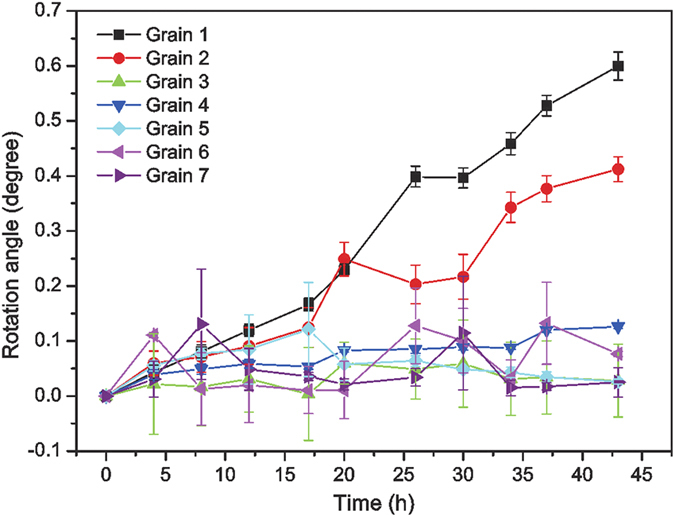
Grain rotation angle as a function of time of all 7 grains. Rotation angles are calculated from crystal orientation matrices, but rotation axes are not shown in this plot. Rotation is induced by the high density of electric current stressing in Grain 1 and 2, but in other grains only orientation fluctuation is observed due to the temperature instability.

The stability of the grain size and morphology provides an opportunity for a more quantitative and detailed study of the orientation evolution. Three identical positions close to the center of each grain are pinpointed in each scan, and the relative rotation with respect to the orientation before EM test at each position is computed, averaged, and exhibited in Fig. 3. Grain 1 and Grain 2, both of which are inside the current crowding region under much higher electric current and current gradient, rotate about 0.6° and 0.4° respectively, well above the angular resolution of μXRD^18^,^19^, and the rotation rate remains almost constant at 0.014° and 0.009° per hour, respectively. From Table 1, we see that the angle between the *c*-axis and the current flow direction of Grain 1 is relatively low, so it is not surprising to see it rotating, because it is known that β-Sn reorients to reduce its resistance under electric current stressing^8^,^9^. However, it is surprising that Grain 2 also rotates, because its *c*-axis is nearly perpendicular to the current direction, which means that its electrical resistivity is already close to its theoretical minimum. Similar to what has been reported previously^8^, grain rotation does not respond to any twinning mode of β-Sn^20^. As expected, the rotation angles detected in this study are much smaller than in the previous one, because of the mild experimental conditions employed here, which provides an opportunity to investigate the onset of the grain rotation phenomenon.

## Discussion

Grain rotation has been unambiguously detected in our study. Previous studies show that the driving force of EM-induced grain rotation in β-Sn is the lowering of the electrical resistance of the system^8^,^9^. Because of the anisotropic crystal structure of β-Sn, the electrical conductivity σ along a certain direction can be calculated as follows^21^:


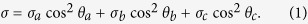


where θ_*i*_ are the angles between the unit cell basis vectors *i* (*i* = *a*, *b*, or *c*) and the electric current direction, and σ_*i*_ denote the electrical conductivities along the unit cell vector directions. Because of the tetragonal symmetry, we have:


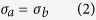


And since:


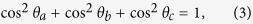


equation (1) simplifies as:


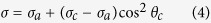


**Figure 4:**
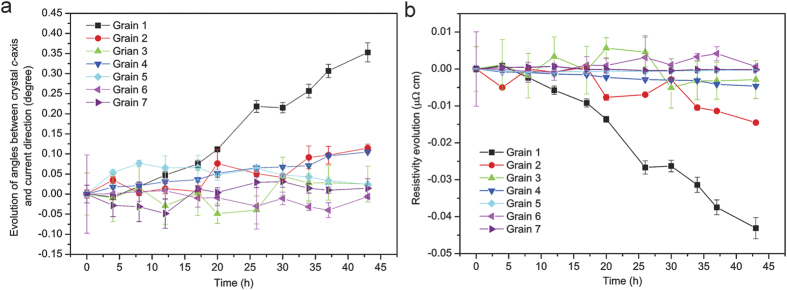
Electrical resistivity evolution resulted from the grain rotation. (**a**) The time dependence of θ_*c*_, which is defined as the angle between the crystal *c*-axis and electric current direction. (**b**) The calculated electrical resistivity σ of all grains. Both θ_*c*_ and σ are found to change in Grain 1 only, although Grain 2 rotates as well.

From eq. (4), it can be seen that the conductivity of Sn is a function of θ_*c*_ only, independent of θ_*a*_ and θ_*b*_. However, measuring θ_*c*_ is not easy because the local current direction cannot be derived accurately from the measurements, especially considering that the depth information is missing due to the bulky shape of the solder joint. Therefore, the evolution of the angle between the *c*-axis of all 7 crystal grains and the global electric current direction (Δθ_*c*_) is measured instead, *i.e.* it is assumed that the electric current direction at any local position of the solder joint does not change as a function of time throughout the performed EM test. This assumption is easily satisfied in this study because the testing condition is mild and no resistance change is observed during the EM test. As displayed in Fig. 4aΔθ_*c*_ goes positive for Grain 1, indicating that Grain 1 has its *c*-axis reoriented more perpendicular to the electric current direction. Interestingly, Δθ_*c*_ of Grain 2, similarly to all other grains, remains close to zero throughout the EM test. In other words, the rotation of Grain 2 is mostly about the *c*-axis, and the angle θ_*a*_ between its *a*-axis and the current direction decreases (Figure S1).

**Figure 5:**
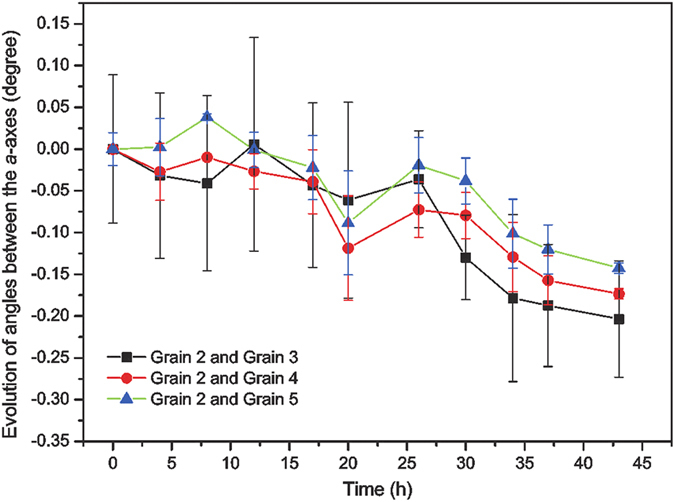
Evolution of the angles between the *a*-axes of Grain 2 and Grains 3 to 5. It shows that the *a*-axis of Grain 2 is reoriented to be more parallel with its neighboring grains.

Using the physical constants obtained from literature (σ_*a*_ = 13.25 μΩ cm, σ_*c*_ = 20.27 μΩ cm)^10^ in eq. (4), the calculated results for resistivities are shown in Fig. 4b. Grain 1 becomes more electrically conductive, which obeys the trend of lowering of the electrical resistivity, while the rotation does not induce any conductivity change in Grain 2, because its *c*-axis is close to a right angle with respect to the electric current direction and its resistivity is already low. To uncover the possible driving force of the rotation of Grain 2, the relative orientation of Grain 2 and its adjacent grains (Grain 3 to 5) are calculated and plotted in Fig. 5. It is found that the angles between the *a*-axes of Grain 2 and all three grains are decreasing during the period of EM testing, perhaps to lower grain boundary energy. Since the self-diffusivity and electrical conductivity of β-Sn are much higher along the *a*-axis than along the *c*-axis, it is easy to understand that the diffusivity of Grain 1 increases as it rotates, resulting in a more significant EM effect deleterious to the Sn solder joint.

**Figure 6:**
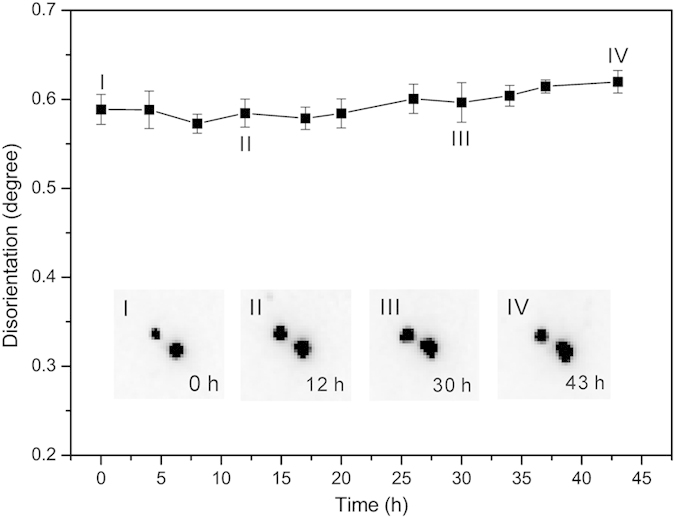
Disorientation angle and peak shape evolution in Grain 1. The disorientation angle between the pair of subgrains and the peak shape keep almost unchanged with time, suggesting the constant density of dislocations in the grain.

Grain rotation is usually achieved via the generation or elimination of GNDs and GNBs, resulting in the variation of dislocation density in a crystal grain or along the subgrain boundaries. To study that effect, we look at the Laue diffraction peak shapes of Grain 1 and Grain 2 from all the 11 scans. Grain 1 is made of two subgrains, as suggested by the pair of subpeaks in the Laue diffraction pattern. First of all, the pair of subpeaks always coexists in all the 11 scans, and the disorientation angle between the pair does not vary with time (Fig. 6). This indicates that the subgrain boundary exists prior to and survives the EM test, and the density of the unpaired dislocations grouped in the subgrain boundary remains constant^22^. Secondly, careful observations of the shape of the subpeaks show that they remain basically unchanged through the experiment. As shown in the insets of Fig. 6, the (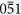) subpeak pair remains sharp when the Sn metal is stressed by the high electric current density. No anisotropic streaking or isotropic broadening of the peaks is detected, showing that the applied electric current does not change the density of either unpaired or paired dislocations in both subgrains^23^,^24^. The case for Grain 2 is simpler, because only one set of Laue peaks is detected, indicating no subgrain boundary. Similarly to Grain 1, no obvious streaking or splitting is observed in all 11 scans. This comprehensive analysis suggests that the microstructure of the GNDs and GNBs does not change through the duration of the electromigration test.

We propose that the observed rigid grain rotation is induced by divergence of the atomic diffusion in anisotropic β-Sn. Grain rotation is detected in the two grains lying within the current crowding region only. There, the electric current density and electron wind force are more than an order of magnitude higher than in the body of the solder joint^12^, leading to faster atomic diffusion. Moreover, the current density and direction in this region are more inhomogeneous, which results locally in highly non-uniform atomic diffusion, in terms of both the motion direction and motion flux. Consequently, such non-homogeneous stress may provide the grains with the necessary aggregate torque for rotation, based on the dynamical theory of diffusion accommodated grain rotation^25^. In contrast with previous observations and simulations in metals with relatively high melting point such as Al and Au, in which grain rotation is accomplished by displacive deformation (dislocation slip and subgrain coalescence)^26^,^26^, our grain-by-grain study of the Laue diffraction peak shape and subgrain disorientation evolution indicates no significant dislocation motion, generation, or elimination in β-Sn. Our finding agrees well with the previously proposed mechanism of β-Sn grain rotation^9^ in which diffusion played the most important role. A recent *in situ* study of the deformation behavior of nanocrystalline Sn pillar inside a transmission electron microscope at room temperature suggests that diffusional deformation could overwhelm displacive deformation when the specimen size is below a critical value^28^. In our study, both Grain 1 and Grain 2 are larger than 100 μm^2^. Considering that β-Sn has a low melting temperature *T*_*m*_ of 505 K at ambient pressure, and that the EM testing temperature is almost 70% of the *T*_*m*_, it is not surprising that the diffusion rate at such high homologous temperature is fast compared to the low strain rate generated by the mild EM testing condition, leading to stress accommodated grain rotation to occur in large grains via diffusion dominated deformation. This finding helps understand not only the onset of grain rotation, but also the EM-induced whisker growth, which is another important reliability concern in Sn solder joints. Because displacive deformation usually takes place relatively quickly and dislocation motion releases stress easily^29^,^30^,^31^,^32^, diffusion is the more favored deformation mode for Sn whisker growth, which requires a continuous and sustained compressive load^33^,^34^.

From the previously reported experimental and simulation work, grain growth is expected accompanying with grain rotation^8^,^9^,^25^,^26^,^27^. The measured decrease of the angles between the *a*-axes of Grain 2 and its neighboring grains agrees with this trend. This is also confirmed by our own experience in a different set of experiments than those described in the present manuscript. When carrying out the EM test at higher temperature (150 °C) on an identical solder joint to accelerate the experiment (Figure S2), grain growth is clearly visible after 28 h.

In summary, taking advantage of the high spatial and orientation resolution provided by synchrotron Laue X-ray microdiffraction, we investigated in real time the grain-by-grain crystal orientation evolution of a Pb-free Sn-based solder joint under EM at the early stage before any resistance change or structure failure is detected. Grain rotation is observed in the two grains within the current crowding region only. Because of the high magnitude and high gradient of electric current density, divergence of atomic diffusion is expected in this region, and thus the stress state in this region becomes highly non-homogeneous, the grain boundary energy becomes unbalanced, and eventually the required aggregate torque for grain rotation is achieved. Furthermore, accompanying grain rotation, the material undergoes diffusion dominated deformation, instead of the more common displacive deformation, and thus no dislocation density and subgrain boundary structure change is detected. The electrical resistivity change resulting from the orientation change is computed, revealing that at this initial stage of EM and with such a small rotation angle, the rule of electrical resistance drop still holds in the grain with high electrical resistivity, while the other grain with low resistivity reorients to have its *a*-axis more parallel with the ones of its neighboring grains.

## Methods

The sample used in this study was from a Pb-free flip chip (Sn−0.7% Cu). The configuration of the flip chip has been described elsewhere^35^. For pretreatment, the flip chip was successively cut into four pieces, ground using SiC sand papers and polished to mirror finish. To stabilize the microstructure and eliminate the residual stress introduced in the polishing process, the sample was annealed at 150 °C for 2.5 h. It was stressed by electric current at a constant average current density of 1.25 × 10^4^ A/cm^2^ at (75 ± 2) °C for 43 h. The test condition was mild and no resistance change was detected during the entire process. Before and during the EM test, the cross-sectioned solder joint was scanned continuously under the microfocused polychromatic X-ray beam on Beamline 12.3.2 at the Advanced Light Source (ALS), Lawrence Berkeley National Laboratory (LBNL)^36^. The angle between the sample cross section and the incident X-ray beam was kept at 45°. The raster step was 3 μm and the exposure time was 0.5 s per point. The X-ray beam was focused to about 1 × 1 μm^2^ using a pair of Kirkpatrick-Baez mirrors and the penetration depth of the X-ray beam within the energy range of 5 to 24 keV in pure Sn was estimated to be about 2–20 μm. At each scanning position a Laue pattern was recorded in reflection geometry using a MAR 133 X-ray CCD detector which was mounted about 8 cm above the sample and 90° with respect to the incident beam. Each scan took about 4 h and contained 3000 Laue patterns, and a total of 11 successive scans were recorded throughout the EM test.

The Laue patterns were analyzed using the software package XMAS^37^. Diffraction peak positions were determined by fitting each reflection intensity profile with a 2D Gaussian function. The diffraction geometry, including the sample-to-detector distance, the center channel on the detector, and the relative tilts of the detector, was first calibrated by indexing a Laue pattern of a strain-free single crystal silicon chip by minimizing the deviation of the angles between the calculated peak position and the experimental data. All the Laue patterns taken on the specimen were indexed using that same calibration. This approach secures high angular resolution (0.01°) for crystal orientation^18^,^19^, which is important for the investigation of the crystal orientation evolution. Furthermore, by studying diffraction peak shapes, information on defects was also obtained, which provides essential clues for characterizing the microstructure of metallic materials. The micron-sized spatial resolution provided by this technique, which is one of the essential differences comparing to diffraction line profile study method^38^, offers an opportunity for grain-by-grain intragranular investigation. This technique has been widely applied in reliability study, not only in solder joints, but also for through-silicon vias^39^ and three-dimensionally printed alloys^40^,^41^.

## Additional Information

**How to cite this article**: Shen, H. *et al. In situ* synchrotron study of electromigration induced grain rotations in Sn solder joints. *Sci. Rep.* **6**, 24418; doi: 10.1038/srep24418 (2016).

## Supplementary Material

Supplementary Information

## Figures and Tables

**Table 1:** The angle between crystal *c*-axis and local electric current direction before EM test.

Grain No.	1	2	3	4	5	6	7	8
Angle (deg)	46.9	84.6	54.9	79.4	87.4	61.3	86.7	69.4
