# Supplementary material for: In situ synchrotron study of electromigration induced grain rotations in Sn solder joints
Source: Sci Rep. 2016 Apr 18;6:24418. doi: 10.1038/srep24418 (PMC4834559; doi:10.1038/srep24418)
Supplement: Supplementary Information [file srep24418-s1.pdf]

In situ synchrotron study of electromigration induced grain rotation in anisotropic Sn solder joints

Hao Shen[1], Wenxin Zhu[1], Yao Li[1], Nobumichi Tamura[2], Kai Chen[1*]

1. Center for Advancing Materials Performance from the Nanoscale (CAMP-Nano), State Key Laboratory for Mechanical Behavior of Materials, Xi'an Jiaotong University, Xi'an, Shaanxi 710049, China
2. Advanced Light Source, Lawrence Berkeley National Laboratory, Berkeley, California 94720, USA

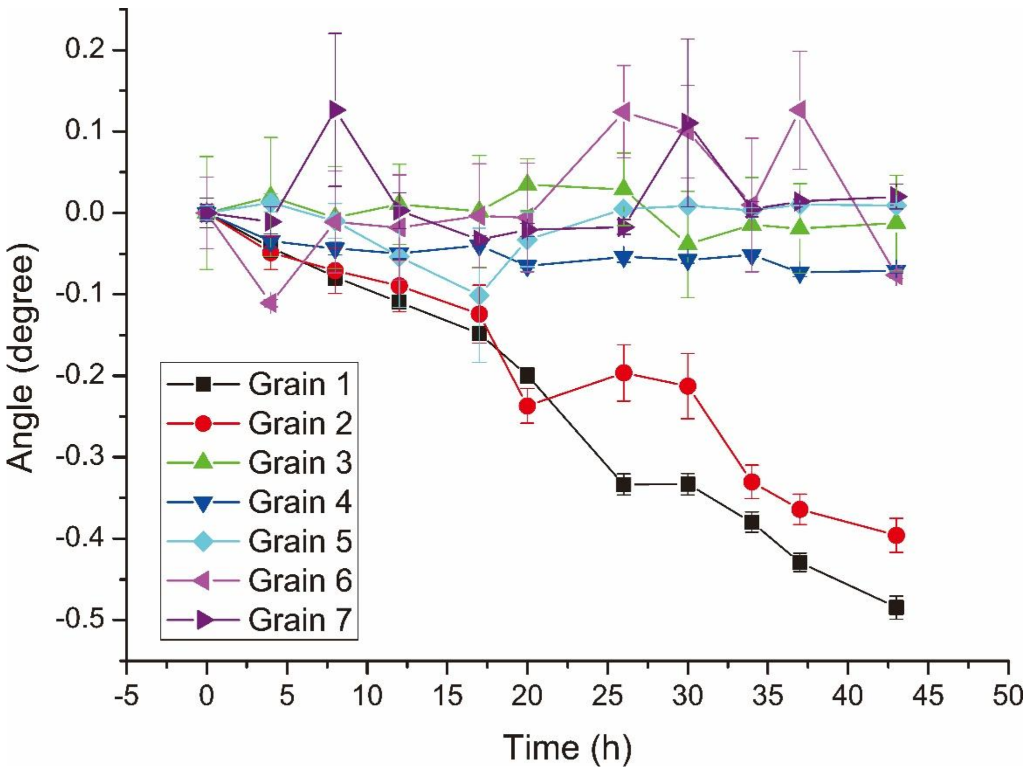

Figure S1. The evolution of θ_a, which is defined as the angle between the crystal *a*-axis and current direction. Comparing with Figure 2 in main text, it is concluded that the rotation of Grain 2 is almost about its *c*-axis.

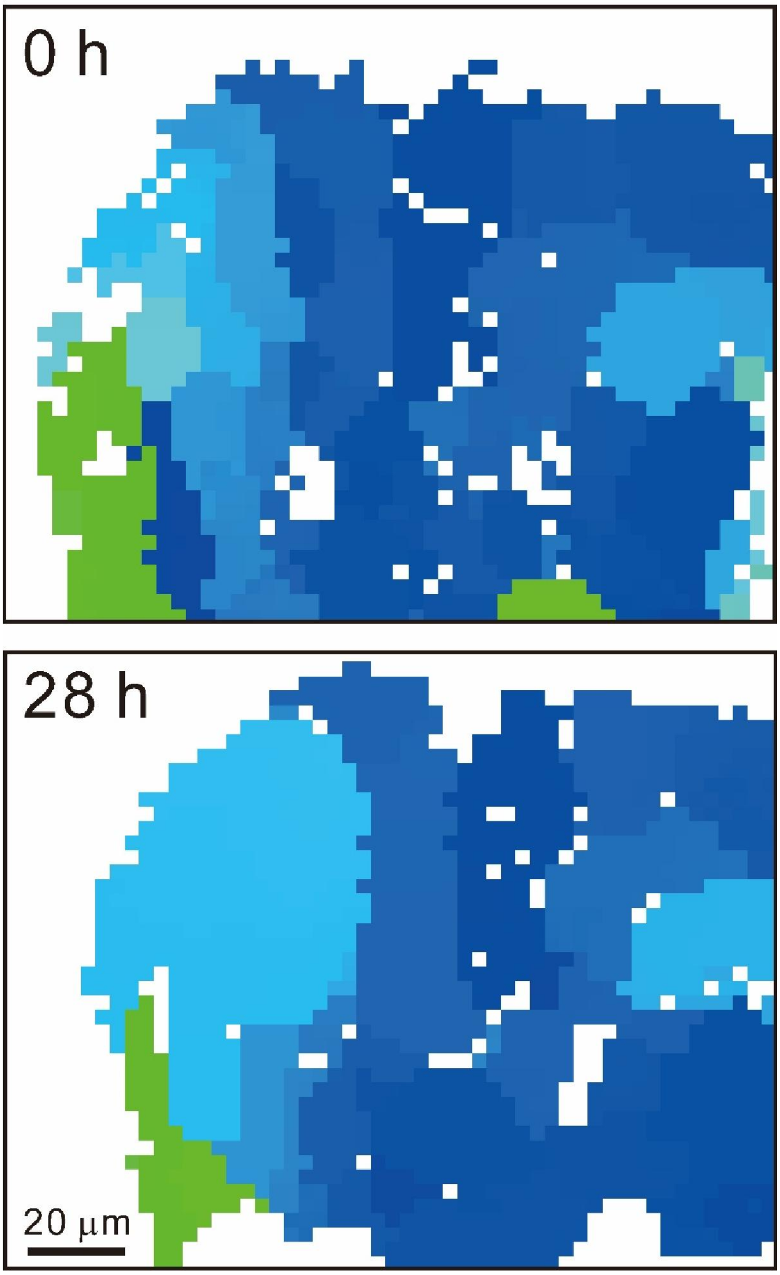

Figure S2. The orientation maps of an identical solder joint before and after 28 h EM test at 150 ^{o}C. The crystal grains grow much bigger in this period.
